# Interviews of community-dwelling older persons in Greece on healthy aging and wellbeing during multiple crises (the HAiG study)

**DOI:** 10.3389/fragi.2026.1787365

**Published:** 2026-04-23

**Authors:** A. A. Mallidou, E. Zioga, A. Magripi, E. Roditi, F. D. Provida, Th. P. Apostolidi, I. V. Papathanasiou, P. Sourtzi

**Affiliations:** 1 School of Nursing, University of Victoria, Victoria, BC, Canada; 2 Department of Nursing, University of Thessaly, Larissa, Greece; 3 Department of Nursing, National and Kapodistrian University of Athens, Athens, Greece

**Keywords:** community-dwelling, Greece, healthy aging, multiple crises, older persons, wellbeing

## Abstract

**Background:**

Healthy aging is a key priority in society, research, policy, and practice. Older people can continue contributing to society if they are healthy, empowered, financially secured, engaged in social activities, and have a sense of purpose.

**Purpose:**

The aim in this study was to explore and understand older people’s perceptions and experiences about healthy aging and wellbeing during multiple crises to support community cohesion and encourage relevant policies.

**Methods:**

In 2023, a convenience sample of 58 older people living in urban and rural regions in Greece were interviewed. A content analysis was performed using the collected data.

**Results:**

Three themes and five subthemes emerged from the data: a. financial stability, where employment was a subtheme for financial stability and security; b. sociopolitical environment; and c. personal choices, which included the four subthemes of healthy lifestyle, family, social engagement, and personal development.

**Discussion:**

Greek older people’s perceptions on healthy aging and wellbeing focused on financial security, the sociopolitical environment, and personal choices for healthy lifestyle and family relationships. The findings indicate the value of implementation of policy initiatives for building a society for all ages and improving older people’s lives and their contribution to society.

## 1 Introduction

The world population was estimated to be eight billion on 15 November 2022 (United Nations Department of Economic and Social Affairs, Population Division, 2022). For the first time in history, older persons (i.e., aged 65 or older) outnumbered the under 5-year-olds and teenagers. In 2050, the number of older people worldwide is expected to exceed 2 billion (16% of the population) and to be more than twice the number of children under age 5 and approximately the same as the number of children under age 12 (United Nations Department of Economic and Social Affairs, Population Division, 2022). Because of mortality reduction, the average global longevity is expected to be approximately 77.2 years in 2050. Therefore, “*To maximize the potential benefits of a favourable age distribution, countries need to invest in the further development of their human capital by ensuring access to healthcare and quality education at all ages and by promoting opportunities for productive employment and decent work*” (United Nations Department of Economic and Social Affairs, Population Division, 2022, pii). It is a myth that older people increase the cost of and obstruct healthcare services since older individuals can live longer and healthier lives, continue contributing to society, and engage socially (United Nations, 2020a; WHO, 2015). In December 2020, the global initiative of the Decade of Healthy Aging 2021–2030 by the United Nations General Assembly (United Nations, 2020b) focused on older persons and planned to bring together governments, professionals, academia, the public, media, and the private sector to build a society for all ages and improve the lives of older people and their families and communities. There is little evidence that older people today are in better health than previous generations. However, strong evidence indicates that good health adds years of life and increases longevity depending on healthy aging that maintains functional ability and enables wellbeing (United Nations, 2020a; WHO, 2015).

Healthy aging is a key priority in society, research, policy, and practice (WHO, 2017; 2021). However, the conceptualization of “healthy aging” and various definitions based on different perspectives and paradigms (Grigg et al., 2018) highlight its multidimensionality and complexity, where providing one inclusive definition is challenging (Menassa et al., 2023).

Healthy aging is defined as “the process of developing and maintaining the functional ability that enables wellbeing in older age” (WHO, 2020). Healthy aging depends on mobility and wellbeing and is inextricably linked to physical activity (WHO, 2023), social engagement, and good relationships with family and friends (Bauman et al., 2016; Hirvensalo et al., 2000). Key determinants of healthy aging also include diet, self-awareness, life-long learning, independence, and sociopolitical involvement (Abud et al., 2022). The WHO’s definition of health as a “complete” state of wellbeing does not include chronic diseases and disabilities because health is defined as the “ability to adapt and self-manage” physical, mental, spiritual, and social domains to maintain wellbeing (Huber et al., 2011) and cognitive functioning. They also state that it is important “*to* e*mpower individuals and communities in order to achieve the highest level of physical, psychological, and social health*” (Jaberi et al., 2019, p. 1552). Health and wellbeing are defined as “*a dynamic balance between opportunities and limitations, shifting through life and affected by external conditions such as social and environmental challenges*” (Huber et al., 2011, p.2).

Wellbeing means that people should be able to fulfill their potential and obligations, manage their independent lives despite disease, participate in social activities, and maintain autonomy and identity (Dröes et al., 2017; Ward et al., 2021). Wellness is a holistic and multifaceted concept defined as “*the optimum state of health and wellbeing that each individual is capable of achieving*” (Hinkle and Drew, 2020, p. 252), which is related to overall wellbeing and health in mind, spirit, and body as a way of life to live fully within the community (Hinkle and Drew, 2020).

Other scholars, though, relate wellbeing with quality of life, which is described as a multidimensional model based on the individual everyday environment (Thompson et al., 2011; Iždonaitė-Medžiūnienė and Preikšaitienė, 2024). However, older adults’ quality of life depends on the stage of their age (i.e., 61–73, 74–85, and >85) (Boustani et al., 2023; Iždonaitė-Medžiūnienė and Preikšaitienė, 2024), which is related to physical health, financial status, social relationships, their personal development, spiritual wellbeing, and intellectual and emotional health (Iždonaitė-Medžiūnienė and Preikšaitienė, 2024).

Healthy aging and quality of life have increasingly become central themes in the literature, with researchers emphasizing their reciprocal and mutually reinforcing relationship. Healthy aging, a multidimensional construct, encompasses the maintenance of physical, cognitive, emotional, and social capacities that underpin quality of life, which fosters the psychosocial and behavioral conditions that sustain healthy aging. These dimensions are widely identified as core determinants of both active/healthy aging and quality of life, each strongly linked to life satisfaction, autonomy, and independence in later-life (Marzo et al., 2023). This perspective aligns with the argument from Gianfredi et al. (2025) that healthy aging arises from the dynamic interaction of biological, behavioral, and social determinants. They underscore the contributions of psychological wellbeing, health-promoting lifestyle behaviors, and supportive environmental conditions to longevity and functional ability. Notably, they highlight evidence showing that positive emotional states and robust social networks are associated with reduced inflammation, improved cardiovascular functioning, and slower cognitive decline—mechanisms that directly connect quality of life to healthier aging trajectories. This multidimensional framework shapes older adults’ quality of life, which in turn influences aging trajectories by informing behaviors, environmental exposures, and psychosocial conditions that promote long-term health. In parallel, Rodriguez and Selvam (2025) conceptualized quality of life through physical, psychological, social, and environmental domains, emphasizing that subjective wellbeing (e.g., emotional stability, cognitive functioning, and perceived social support) plays a critical role in shaping health behaviors and aging outcomes. They argue that quality of life is not merely a consequence of health status but an active determinant of healthy aging through behavioral and psychosocial pathways. Additionally, healthy aging and healthy longevity are used interchangeably to describe the healthy aging process of individuals (Zheng, 2022a). The dramatic increase in the oldest-old population (i.e., >85 years) may increase the need for healthcare services compared to younger old adults (i.e., <80 years).

In China, the continuous improvement of social conditions, gradual development of the policy system, and the rapid economic growth of the country resulted in improvement of social welfare programs, which changed the living conditions and lives of older people in both urban and rural areas of the country (Zheng, 2022a). There is a positive effect of socioeconomic status (SES) and socioeconomic context (e.g., political background and family and cultural structure) on an individual’s health, including older adults (Li and Xia, 2022). That is, the higher the SES of a person, the better their health status. However, people with poorer health are less likely to achieve higher SES and higher income (Li and Xia, 2022). Additionally, people with lower living conditions and SES experience more crises and uncontrollable and adverse life events (e.g., crime, violence, discrimination, illness, or death of a child) (Li and Xia, 2022). Loneliness and social relationships also affect health. Opportunities to participate in social life, accumulation of experiences, and sense of control over life influence later-life patterns and play a role in the aging process when people reach a certain degree of material living conditions (Li and Xia, 2022). Social participation and pension influence healthy aging and older people’s quality of life in rural and urban areas (Zeng, 2022b). Zhang and Wei (2022) contend that the prevalence of disability among the urban oldest-old population in China, measured by the presence of at least one limitation in the activities of daily living (ADLs), is higher than that observed in rural areas. They argue that the comparatively stronger allocation of healthcare resources in urban settings enables very old adults to survive longer while living with disabilities. In contrast, healthcare services in rural regions often struggle to meet the needs of older people with relatively poor health conditions (Zhang and Wei, 2022). At the same time, improvements in living conditions, infrastructure, socioeconomic development, and overall standards of living in rural areas have contributed to better general health among older adults (Zhang and Wei, 2022).

The Greek population is aging rapidly; especially, the population in the rural regions of the country (Eurostat, 2025a) is aging rapidly due to rapid declines in both fertility and mortality. At the time of data collection, Greece was in the midst of a lengthy economic, politico-social, cultural, and health crisis following the 2009 international recession. Older persons were the group most vulnerable to the consequences of these crises, which influenced their wellbeing. A recent visit of the first author to Greece provided the opportunity of discussions with various older persons who shared their lived experiences that are relevant to healthy aging. These discussions inspired the idea to explore and capture older people’s experiences on healthy aging and wellbeing and compare the data from before and during these crises. This study is associated with the unique natural experiment of several crises that occurred in Greece for 13 years (2010–2023) that have transformed the society and turned it back by decades (Eurostat, 2025b). Understanding of Greek older people’s experiences under those unpleasant situations and harsh economic conditions is crucial in supporting democracy and transparency for sustainable development and cohesion in society.

This study’s primary *purpose* was to capture the *perceptions* on healthy aging and wellbeing of community-dwelling older people in Greece and their *lived experiences* during multiple crises such as economic (e.g., international recession), political (e.g., policies that suppress vulnerable population groups such as immigrants, older people, and poorer people), social and demographical (e.g., massive and elevated rates of immigration of the young generation), cultural (e.g., change of priorities, needs, and preferences), and health (i.e., the COVID-19 pandemic) from 2010 to 2023. Our main objectives were to 1) understand and capture the potential association of economic and sociopolitical conditions with older peoples’ wellbeing; 2) support collective discussions and community cohesion; and 3) use the data and findings to influence decision-makers for developing relevant healthcare policies.

## 2 Materials and methods

### 2.1 Study design

From a constructivist point of view, this exploratory descriptive qualitative study (Liow et al., 2022; Flanagan and Beck, 2025; Patton, 2015; Thorne, 2013), a distinct methodologic classification in its own (Sandelowski, 2010), was conceived, designed, and conducted in collaboration with a non-profit organization, namely the Hellenic Association of Gerontology and Geriatrics (HAGG), two academic institutions in Greece, and a Canadian university. Using the term *constructivist*, we “acknowledge subjectivity and the researcher’s involvement in the construction and interpretation of data” (Charmaz, 2014, p. 14). This qualitative design is well suited to eliciting rich and contextualized descriptions and facilitating cross-regional comparisons of themes emerging from participants’ narratives (Flanagan and Beck, 2025).

### 2.2 Settings and sample

The *target population* consisted of older persons living in urban or rural communities in Greece. We selected this population based on a local rumor that, during multiple crises, older people living in urban areas experienced more severe consequences than those living in rural areas. We anticipated that the collected data would enable a comparative analysis of the emerging themes across these distinct regions of the country, with the results to be disseminated in a subsequent publication. We had also planned to recruit eligible participants living on the island of Ikaria. Ikaria is recognized as a Blue Zone, where residents exhibit exceptional longevity and comparatively healthy aging profiles. However, due to weather-related disruptions (e.g., multiple flight cancellations during the data-collection phase), travel to Ikaria was not feasible. Our *accessible population* resided within urban or rural communities in the Greater Area of Athens (Attica) and Thessaly, Greece, respectively. Using a convenience (volunteer) sample of knowledgeable, articulate, and reflective informants (Flanagan and Beck, 2025) within each region, we selected eligible participants based on predefined inclusion and exclusion criteria to ensure that the sample aligned with the study objectives. The eligibility criteria were as follows:

- Older adults aged 75 years or older to capture the perspectives and lived experiences of individuals in later life.
- Residents of the selected regions, ensuring cultural and contextual relevance to the study’s focus on geographic variation.
- Able to communicate in the Greek language, in order to participate meaningfully in the interview process.
- Cognitively capable of providing informed consent, as assessed informally at the start of recruitment.
- Willing to participate in and able to allocate the required time for an individual interview.

Individuals were excluded if they had significant cognitive impairment that prevented informed consent, were unable to participate in an interview due to severe functional limitations, or did not meet the language criteria.

Over 100 eligible older adults expressed interest in participating in the study and sharing their experiences and perspectives on healthy aging. However, due to limited resources, we were only able to conduct a total of 58 individual interviews, prioritizing scheduling on a first-come, first-served basis.

### 2.3 Data collection

Based on the literature (Hennink and Kaiser, 2022; Velaithan et al., 2024), we developed a *guide interview survey* on participant demographics, healthy aging, and wellbeing during three periods: before 2010, 2010–2020, and 2020–2023. Between January and August 2023, interested and eligible participants were contacted by our research assistants, who explained the proposed study, answered questions, and assisted the potential participants to sign the consent forms. Then, the research assistants conducted in-depth, face-to-face, semi-structured *individual interviews* (approximately 60 min each) at times and locations that were convenient for participants (e.g., their homes), indicating a study in various natural contexts (e.g., participant homes). All research assistants completed the required institutional research ethics training (e.g., TCPS 2 or equivalent) prior to engaging in data collection. Additionally, the research assistants underwent comprehensive training led by the principal investigator, emphasizing qualitative interviewing with older adults and methodological rigor. The training included the following subjects:

- Orientation to study aims and qualitative paradigm (e.g., epistemological assumptions underlying semi-structured interviewing, the role of researcher positionality and reflexivity, awareness of potential biases, the importance of attending to non-verbal cues, pacing the interview, and creating space for participants to shape the direction of the conversation).
- Qualitative interviewing techniques (e.g., strategies for building rapport, using open-ended and follow-up probes, facilitating narrative depth, managing emotional and sensitive topics in a supportive manner, attending to non-verbal cues, pacing and respectful closure, and role-playing exercises).
- Ethical considerations (e.g., best practices for obtaining informed consent in a conversational manner, safeguarding confidentiality during in-home interviews, responding appropriately to participant distress, ensuring participant autonomy throughout the interview, and cultural humility).
- Practice and calibration (e.g., mock interviews and iterative feedback to promote consistency in tone, depth, and neutrality).
- Data management procedures (e.g., secure audio-recording, field-note documentation of contextual and observational field notes, maintaining detailed reflexive memos immediately for each interview, file naming, de-identification, and secure storage and transfer procedures).
- Ongoing supervision (e.g., periodic team debriefings and case consultations to troubleshoot challenges, enhance reflexivity, and support consistency in data collection across research sites to maintain protocol fidelity).

Interviewers took written notes and audio-recorded or videotaped the interviews, depending on the participant’s consent. Recruitment continued until data saturation was reached, at which point no new themes emerged (Hennink and Kaiser, 2022). Saturation was assessed through ongoing review of the codebook breadth, depth of accounts, and redundancy of insights across subgroups and regions. The principal investigator reviewed transcripts for accuracy, and they stored the collected data (i.e., video and audio recordings, transcripts, and notes) in encrypted and password-protected computer files within a locked cabinet in their office. De-identified data (i.e., transcripts only) were shared with the research team members using encrypted and password-protected computer files for analysis purposes only.

### 2.4 Data analysis

In this naturalistic descriptive qualitative study, which used an iterative process and reflexive inductive approach, a thematic analysis (Braun and Clarke, 2022) was conducted for identifying conceptual patterns and meaningful themes emerging from the data. This process supports both inductive interpretation and rigorous, transparent documentation of analytic decisions (Flanagan and Beck, 2025). Data collection and data analysis were carried out concurrently. The research team immersed themselves in the data by reading the transcriptions repeatedly and worked collaboratively on analytic tasks toward achieving a consensus. In each setting (i.e., Thessaly and Attica), under the supervision of a researcher, two research assistants *independently* collected, transcribed verbatim, provided a code number to each participant, analyzed the transcripts, and developed their own thematic codes and coding structure to increase credibility. The principal investigator independently analyzed and coded the transcripts of all the interviews across both settings (n = 58). Then, the initial codes generated across the dataset were compared and used to finalize the coding structure through discussion with the principal investigator and a third researcher (i.e., the supervisors of research assistants). The relationships between codes were evaluated, and themes were determined. We thoughtfully used our own experiences and acquired knowledge to enhance the sensitivity of the themes that emerged. We maintained reflexivity through data analysis by recording, discussing, and challenging established assumptions based on the research assistants’ reflexive diaries. Without using computer-assisted qualitative data analysis software, we developed an analytic matrix to identify patterns and connections among the emerging themes and audited the thematic analysis. The rationale for using a manual method of data analysis, instead of computer-assisted, is twofold: a) it allowed us to get closer to the data and b) the time spent learning the software, regardless of its many advantages, may reduce the time available for actual analysis (Flanagan and Beck, 2025). To ensure rigor and increase the authenticity in our methods, we used audit trails, multiple coders, investigator and data triangulation, peer debriefing, and member checks that supported the trustworthiness (i.e., credibility, dependability, confirmability, and transferability) and validity of the analysis (Lincoln and Guba, 1985). A member check was performed with several participants through an in-person presentation of the preliminary findings and discussion of the major themes. Finally, we reviewed the data for any different participant perspectives that did not fit into the themes that emerged (goodness-of-fit). The present paper focuses on reporting the descriptive findings related to healthy aging and wellbeing along with the participants’ primary concerns and recommendations.

### 2.5 Ethics approval

The HAiG study was conducted in accordance with the guidelines of the Declaration of Helsinki. The Human Research Ethics Board at the University of Victoria approved our study (#22-0480/23Aug2022). Operational approvals were obtained by the local authorities in Greece, namely the National and Kapodistrian University of Athens (#422/18Nov2022) and the University of Thessaly (#1521/21Oct2022), where the data were collected. Respondents provided written informed consent to participate in the study before starting the interviews. Their informed consent included communication and comprehension of information, voluntary participation, and agreement that the study was to be published including only anonymized information. We had neither relationships nor interactions with the participants, nor did their personal characteristics, qualifications, or perspectives influence the methods, results, or transferability of the findings (reflexivity).

## 3 Results

The primary purpose of this study was to capture the perceptions to healthy aging and wellbeing of community-dwelling older people in Greece, along with their lived experiences during multiple crises such as economic, political, social, health, and cultural. In this paper, we only report the primary findings regarding healthy aging and wellbeing and participants’ primary concerns and recommendations for healthy aging and wellbeing.

### 3.1 Descriptive characteristics of participants

The study participants were mainly women (n = 33 or 57.89%), as expected; they were aged 72–95 years (mean: 81.16 years old); were married/common law (n = 35 or 61.40%) or widows (n = 17 or 28.82%); and had 0–5 kids, 0–14 grandkids, and/or 0–5 grand-grandkids (mean: 2, 3, and 4, respectively). Their monthly income (mainly pension) ranged from €0 to €1,750 (mean: €1,210), but 21 (approximately 37%) of participants’ monthly pension was less than €500. Most respondents reported that they share love and support with only “a few” close friends and “some” family members.

### 3.2 Themes that emerged

Through the data analysis and subsequent discussions, we identified three themes and five subthemes across all 58 interviews that emerged from the data related to participants’ lived experiences and perceptions of healthy aging and wellbeing. All the identified themes are related to a dynamic, interactive, multidimensional system in which *financial stability* and employment, *socio-political environment*, and individual *personal choices* (i.e., healthy lifestyle, family, social engagement, and personal development) shape the pathways to wellbeing.

#### 3.2.1 Financial stability

Following the first interview, financial burden emerged as the most significant theme and employment concern, which was evident from the frequency with which the participants raised the issue and the extensive comments they provided. They expressed despair about the increasing cost of living across both regions (i.e., Thessaly and Attica). In the participants’ own words:

- “I am retired with a more than 90% reduction of my income” (P34)^1^; “my pension has been reduced by 45%, while I was planning to live a comfortable life during my retirement” (P15); my/our income was reduced drastically (about 75%!) (P16, P42, P43, P44, P45, P48, P51) that brought a lot of problems (P53, P55).
- Having a regular work to put food on the table every day (P12, P13, P14, P15) without debt is important for financial independence (P12, P13, P14, P16, P47, P51, P54, P57, P58).
- “I had no financial or other problems before; now things are difficult financially” (P12); the quality of life changed to the worst financially and socially (P13 and P32); cannot afford the necessary needs for a decent life such as food, children education; only the wealthy people can afford the living cost (P14, P15, P34, P37, P46, P49, P50, and P58); everything is so expensive, the super-market food prices have been doubled (P14, P35, P49, P50, P51, P56, and P58); we have to count every penny (P14 and P16); cannot buy medications without my kids’ assistance, other benefits declined completely too, we cannot afford living (P43 and P44); health has deteriorated since treatments are unaffordable (P50, P54, and P57); “rent increased and utilities too, which made us so upset; I am in despair” (P45).

At the same time, though, some participants did not highly rate the importance of money. In their words, “Money is not the ultimate good in life” (P6); “creating wealth does not ensure happiness and a peaceful life” (P32); “avoid luxurious life/things” (P44); “money makes life easier, but do not sacrifice personal values for money’s sake” (P6, P32, and P44). “Careful financial management (P19 and P51) and spending wisely (P24, P26) are essential factors for a household to survive today; and everything will go as it should” (P34).

#### 3.2.2 Sociopolitical environment

The participants’ voices were consistent regarding the sociopolitical structural conditions and expressed their indignation for an unfair political situation. The following are their quotes:

- “Our authoritarian government” (P18) “does not care for us, the citizens” (P32). “I hope that my mate citizens will vote for a moral not corrupted government” (P15). “There are plenty homeless people and ‘syssitia’ (i.e., free meals provided by the church or charity organizations) in Greece that we had never experienced it before” (P14). “We experienced lower income due to the government’s decisions; I am still upset with the current situation and economics; we worked hard to develop wealth, we did not steal anybody’s money, we did not upset anyone… we want our stolen lives back” (P20). “Nothing is certain, depending on the economic bargain at the international level, where the game is played and the greedy rich becomes richer and the poor, poorer as they exploit the poorest” (P5).
- During COVID-19, “it was very difficult; a type of dictatorship, even worst, because you could not even go outside of home” (P46). “I served my penalty at home, because I felt like a prisoner” (P12); “we were afraid that would be the next victims of the pandemic” (P15).

Study participants also argued that health and employment within acceptable working conditions are among the basic human rights that the state must guarantee for all citizens. The following are their quotes:

- Being employed regularly (P24, P29, P30, and P31) “in decent working conditions” (P28) is vital for wellbeing.
- The government must take measures to improve our everyday lives, invest in citizens’ health and wellness (P13, P14, and P15), and stand by the young people to avoid emigrating for various reasons (e.g., limited jobs and social injustice) (P14 and P58).
- “Otherwise, Greece will need many years to overcome these multiple crises” (P14).

#### 3.2.3 Personal choices

The participant’s various perceptions on healthy aging are summarized as a multifactorial concept and achievable goal that relies on personal and familial healthy lifestyle choices, family relationships, social engagement, and personal development. In their general statements,

- Healthy aging means to feel and perceive that you are still young and enjoy it (P11, P41, and P50); “have an active life” (P47) and hobbies that provide satisfaction and meaning to life, energy, productivity, and creativity (P15, P16, P17, and P18); live life to the fullest (P14 and P47) “with dignity, decency, and respectability” (P58).
- “enjoy the moment and life as it comes and avoid worrying about everything” (P34), and “never complain” (P39); “be happy and proud of yourself” (P12) because “happy people do not get sick” (P34).

##### 3.2.3.1 Healthy lifestyle

- The participant’s “recipes” for healthy aging, wellbeing, good life, and happiness include diet (P9, P10, P11, P12, P15, P18, P20, P39, P40, and P47), regular exercise, healthy habits, and optimistic and modest lifestyle. They quote:
- Walking every day, exercising regularly, dancing frequently (P9, P12, P17, P18, P36, P37, P40, and P47), and enjoying small things in life contribute to facing health issues (P34 and P58).
- When young, personal choices and discipline to healthy habits are important (P12, P18, P15, P20, P32, P47, and P57) “to prevent diseases and control over body and mind” (P32).
- Be self-sufficient, self-serviced, and self-contained for physical and cognitive health (P15, P37, and P58); be optimistic, self-disciplined, and frugal (P11 and P12); “continue living normally after retirement, and do not quit easily from life” (P17).
- Being physically, psychologically, spiritually, and mentally healthy with intact cognition, i.e., “eyes to see and mind to think” (P31), is essential (P11, P12, P13, P14, P16, P17, P18, P19, P31, P34, P35, P36, P46, P47, P51, P55, P56, and P57).
- Physical health is important; take care of yourself every day (P15, P16, P17, P19, P31, P34, P37, P46, P47, P56, and P57), and “courageously face life difficulties” (P16).
- Living a “minimalistic or modest life (παν μέτρον άριστον)” (P16), having a stoic stance in life (e.g., happy to be alive) (P13, P17, and P42), and enjoying the small things (e.g., drinking coffee with spouse in the morning and going out with friends) (P34, P37, P39, P44, and P48) are essential.
- Avoidance of stress (P10 and P53), smoking, drugs, excessive alcohol consumption, and staying up too late in clubs (P9, P15, P40, P41, P44, P48, P49, P54, and P55) were the most common recommendations to the younger generation for healthy aging.

##### 3.2.3.2 Family

Family life with many children and friends, maintaining healthy relationships, and having regular fun is a pattern of healthy aging that was described by most of the participants. The following are their quotes:

- “Choose carefully a partner/spouse who understands and supports you” (P49) “and take care of each other regularly” (P12). In the long term, a happy marriage and joyful moments with the spouse, family, and friends are key for wellbeing (P14, P33, P34, and P37) and “for receiving love and support” (P35). “Sexual activity is important too for energy, strength, and satisfaction” (P12).
- Create a *family* with many children (P5, P7, P29, and P53); the ultimate happiness is to “enjoy healthy kids and grandkids” (P20), sing with them every day (P36, P39, P52, P53, and P57), and have fun regularly (P34, P39, P40, and P58); plan trips to open the mind and create pleasant memories (P14, P34, P39, and P58) and see them thriving in life (P17 and P35) or getting married (P33 and P37).
- Families of at least three generations should stay closely together for kids to learn from grandparents and be grounded in life (P18 and P11).
- “Maintain a pleasant home environment” (P16) and maintain family cohesion to avoid loneliness (P14 and P15). “Support, take care, and love them unconditionally” (P19) as love is “the greatest glue among people, especially within a family” (P14); they may return love and support later (P41 and P44). Maintaining healthy relationships with spouse, family, and friends (P20, P32, P33, P37, P47, P54, and P57) is key for wellbeing.

##### 3.2.3.3 Social engagement

Social life and regular social engagement are essential ingredients for wellbeing. The participant’s describe them eloquently:

- Have a social life and good friends (P37 and P58) to “maintain social connection and cohesion” (P16). Treat others with respect and be polite; love everyone (P16, P55, and P32) and share best practices with neighbors, fellow citizens, and friends to get satisfaction from contributing to the community and changing society (P16, P20, and P36).
- Life-long learning is key for awareness on the prevention of diseases and sickness (P12) and for health promotion (P15, P18, and P19). “The more you read, the better you become. Even in my age when I travel, I take 5 books with me to read, to keep my memory working and to be calm” (P54). “I try to take a new certificate every year by attending online free courses for keeping my mind functional” (P3). “When you find an opportunity, get knowledge not degrees; knowledge and experience improve your personal development” (P5).
- Believe in solidarity (P2 and P6) and “step into the shoes of others to make society better” (P6).
- Be socially engaged and make efforts for social cohesion (P10 and P25) and collective wellbeing (P13 and P6).
- Have a social life (P10 and P45), sociopolitical involvement, and tirelessly work for social cohesion and collective wellbeing (P6, P10, P13, P18, and P25). Having good friends (P10, P44, P48, P51, and P55); “flirting regularly and living a simple life are keys for wellbeing” (P10).

##### 3.2.3.4 Personal development

Participants provided advise for youth on how to live a decent life, full of dreams and actions toward a better life and personal development that, in turn, may lead to healthy aging. The following are their quotes:

- “Be diligent while you are young, so that you do not regret it later” (P19). “Demand, demand, demand the best for your life! For better democracy and social justice. Resist illogical and stressful situations. Do not accept what is offered and weaken us, the citizens. It is our individual responsibility to improve society, because society is us! If we demand it, those who govern will be forced to do it and adapt to our demands” (P6).
- “Be an explorer of life (like goats), not a sheep that follows others; make own decisions for ethical and honesty life” (P18).
- Have a vision and purpose in life (P9 and P17) and “honest relationships” (P17). “Know yourself; develop own abilities to the maximum extent possible in a climate of harmony” (P25). “Be yourself and self-confident” (P2) and a critical thinker to avoid brainwashing (P2 and P6). Do whatever you want to be happy; follow your heart (P13 and P53); set goals and pursue them (P6, P57, and P58); have positive thoughts, and be hopeful and optimistic (P6 and P57).
- Be informed, but wisely use technology (e.g., cell phone and internet) and “avoid watching too much TV” (P15).

## 4 Discussion

Our aim was to capture the perceptions on healthy aging and wellbeing of community-dwelling older people in Greece, along with their experiences during multiple crises. The results provide key messages for actions and policies to support healthy aging and wellbeing categorized into *financial stability*, *sociopolitical environment*, and *personal choices*.

### 4.1 Financial stability

The 2010 crisis caused many burdens (e.g., recession, economic instability, and income reduction) that negatively influenced everyone’s life, which was worsened due to the retirement of many participants during that period. Finance and economic instability were one of the most significant problems and concerns expressed by participants, emerging as the most prominent barrier to healthy aging and wellbeing. Participants described how limited income and fluctuating expenses, particularly costs related to healthcare, forced them to make difficult trade-off decisions, such as choosing between purchasing prescription medications or buying groceries. This finding is consistent with that of Robles et al. (2023) showing that financial insecurity and unstable economic conditions pose major challenges to healthy aging, often functioning as the primary barrier to maintaining physical, mental, and social wellbeing.

Targeted policy interventions can reduce socioeconomic disadvantages and promote healthy aging (MacGuire, 2020). Considering the lifelong hardships that this generation has gone through, they perceive financial security as a determinant and essential feature of healthy aging (Abud et al., 2022). Financial security is associated with health (Burton et al., 2021), the ability to maintain quality of life, including social interactions (Cruwys et al., 2019), affordability for proper care with aging (Hui Chian Teh et al., 2020), fear of needing a caregiver at home or going to a nursing home, and concerns of not needing financial support from their children or not being able to provide financial assistance to their families (Wiggin et al., 2021). In our study, the participant’s concerns include the aftermath of the crises, such as facing challenges in providing necessary financial support to their children. Therefore, providing economic security among older people enables wellbeing not only through fulfilling their basic needs but also by creating crucial social settings for upholding health and resilience (Cruwys et al., 2019). Strategies to support healthy aging include expanding access to affordable housing to enable aging in place, along with strengthening social protection systems to address socioeconomic gaps and ensuring adequate minimum pensions (Caristia et al., 2026).

### 4.2 Sociopolitical environment

The physical (e.g., access to support services, walkable streets, and green spaces) and sociopolitical environments (e.g., income, democracy, justice, homes, neighborhoods, and communities) are closely associated with healthy, active, and successful aging (Menassa et al., 2023; Belachew et al., 2024) and quality of life (WHO, 2025) regardless of the existing variations in older people’s genetics and personal characteristics (e.g., sex, ethnicity, and socioeconomic status). The environments that people live in as children, or even as developing fetuses, have long-term effects on how they age (WHO, 2025). Physical and social environments (e.g., availability of safe and accessible public buildings and transport and places that are easy to walk around) can affect health and healthy behavior (e.g., eating a balanced diet, engaging in regular physical activity, and refraining from tobacco use) that contribute to healthy aging (WHO, 2025). These environments are also risk factors for mortality and functional decline in older age (MacGuire, 2020). During national crises and economic hardships, people tend to experience depression, which affects their emotions and increases worries about future generations (Okun and Ayalon, 2024). Hernandez et al. (2025) argued that healthy and accelerated aging are associated with lower income and other adverse factors, including social (e.g., socioeconomic and gender equality and migration) and sociopolitical (e.g., political representation, free political parties, inclusive suffrage, credible elections, and local democracy) determinants. They also found that accelerating aging was more evident in Eastern and Southern Europe, with considerable disparities across nations.

The Government of Canada’s National Seniors Council (2025) emphasizes that healthy aging is shaped by age-friendly environments, social inclusion, and community support. Their framework highlights the structural determinants of both healthy aging and quality of life, arguing that accessible transportation, safe housing, and inclusive community design are essential for maintaining functional ability and social participation. This policy lens expands the discussion beyond individual behaviors to the broader systems that enable or constrain aging trajectories.

The sociopolitical environment in Greece is currently characterized by a stable right-of-center government, which is making efforts to improve the economic indicators and social challenges regarding living standards and housing (OECD, 2025). However, public discontent remains high due to the rising costs of living, inequality, and concerns over democratic procedures and the rule of law (Rosalux, 2024; Greek Reporter, 2025; OECD, 2025). A significant portion of the population faces financial strain due to low wages relative to high inflation, energy costs, and a skyrocketing housing crisis, particularly in Athens (OECD, 2025). Approximately 27% of the population is at risk of poverty or social exclusion (OECD, 2025; Freedom House, 2024). Youth unemployment remains among the highest in the EU, and women continue to face significant obstacles in the workforce, with one of the lowest employment rates in Europe (OECD, 2025). Over 500,000 mostly young and educated people have emigrated since 2009, contributing to a shrinking and aging population (OECD, 2025).

### 4.3 Personal choices

In Greece, older generations grew up during the difficult socio-political and financial times of WWII and the post-war period; , they also experienced mass migration abroad and a dictatorship. The current older generations learned to practice personal responsibility in all aspects of their lives to successfully survive (Kontzamanis, 2017). Although our sample is not representative of all Greek older people, personal responsibility is an expected finding that the younger generations have been advised to pay attention to (Wiggin et al., 2021; Whitmore et al., 2023), along with taking self-care actions to maintain and promote health (Seah et al., 2021). Healthy lifestyle habits (e.g., Mediterranean diet) and their associations with health outcomes and quality of life in older age are well known (Huang et al., 2025), indicating promotion of healthy aging and prevention of age-related diseases (Mazza et al., 2021).

Older people perceive healthy aging as active civic engagement (e.g., social and political participation) for social wellness and good health (Wiggin et al., 2021; Nyqvist et al., 2024), which affects their health, meaning, purpose in life (Serrat et al., 2017), and cognitive functioning (Tough et al., 2017; Posis et al., 2024); “the power of unity strengthens us” (Okun and Ayalon, 2024, p. 806). Social participation (e.g., volunteering or caregiving) affects wellbeing (Serrat et al., 2017; Okun et al., 2013), while political participation (e.g., campaigns and protests) aims “directly or indirectly, at influencing political outcomes, changing the institutional premises for politics or affecting the selection of personnel or their choices” (Nygård and Jakobsson, 2013, p. 67). Politically active older people have higher levels of wellbeing (Serrat et al., 2017; Okun and Ayalon, 2024); they have a sense of democracy when their rights and voices are heard (Nyqvist et al., 2024), reduction of loneliness (Okun and Ayalon, 2024), expansion of their social circle, and a sense of security originating from belonging to a group of people who share the same values, social solidarity, and moments of hope and optimism; they are empowered, happier, and more satisfied with life (Okun and Ayalon, 2024), which is linked to wellbeing (Serrat et al., 2017; Nyqvist et al., 2024). Higher levels of social trust in a country indicate trust in the system and may discourage active political engagement among older adults (Nyqvist et al., 2024).

Zeng (2022b) described that shifts in family-related cultural norms in China have weakened the traditional role of families in providing emotional support, leaving many older adults increasingly vulnerable to mental health challenges. As a result, they face heightened risks of cognitive decline and various psychological disorders. Without effective preventive strategies, the growing incidence of these conditions is likely to impose substantial burdens on both families and society (Zeng, 2022b). Given these trends, conducting rigorous research on the cognitive functioning and wellbeing of older adults, along with the factors that shape these outcomes, is crucial for promoting healthy aging and enhancing the quality of life for a rapidly expanding older population.

### 4.4 Limitations and strengths

The study limitations and strengths are intertwined. First, the study design as descriptive qualitative research is a limitation in terms of the depth and broadness of data collection and analysis. Second, the specific and narrow geographical area of the study settings (two regions within one country) may influence the transferability of the findings. Third, the convenient non-representative (limitation) but large (n = 58) and geographically diverse sample (strength) provided depth and various perceptions on healthy aging and wellbeing due to the different cultures and customs across the regions and contributed to the findings’ transferability (Abud et al., 2022). However, the characteristics of the participants (e.g., age and education) may be a limitation to the study. Fourth, overrepresentation of women in our sample might potentially have influenced the results (limitation), but gender differences in perceptions were not identified in the data. Fifth, participants’ reluctance to directly respond to sensitive questions related to politics, economic status, and the sociopolitical environment indicates that multiple crises had an impact (strength) on participants’ wellbeing and quality of life that they hesitated to discuss with interviewers. Finally, another strength of the study is the collaboration with the HAGG, researchers, and emerging scholars (i.e., graduate students) across two countries and three universities.

### 4.5 Implications and recommendations

The findings provide important implications for practice, policy, and research. For *practice*, healthcare professionals may benefit by incorporating social determinants of health into their practice with older people since functional ability, an age-friendly environment, and the economic situation influence healthy aging and wellbeing. For *policy*, decision-makers can transform society by achieving the Sustainable Development Goals related to older persons’ health and wellbeing and by implementing practical lifestyle-specific interventions focusing on modifiable factors (e.g., physical activity, nutritional diet, financial security, and social cohesion) for improving healthy aging at the individual level. For *future research*, the influential role of social support on healthy aging should be explored further and evaluated in a broader geographical area (e.g., other European countries) using a different study design (e.g., cross-sectional/national) with participants from various age groups of the population. Our research team is working on additional and more comprehensive analyses and deeper insights regarding older adults’ resilience, vulnerabilities, and support needs that will be disseminated in subsequent publications. A comparative analysis of wellbeing across the periods before 2010, 2010–2020, and 2020–2023 will be addressed in a separate manuscript given the substantial detail and significant themes that emerged from the analysis.

## 5 Conclusion

During multiple crises, Greek older people’s perceptions and lived experiences focused on financial security, the sociopolitical environment, and personal choices for healthy lifestyle and family relationships. Insights could guide meaningful actions by policymakers, the government, and researchers to design and implement policy initiatives for social change to support healthy aging and wellbeing. Building a society for all ages can improve older people’s lives, who can continue contributing to society if they are healthy, empowered, financially secure, engaged in social activities, and have a sense of purpose.

## Data Availability

The raw data supporting the conclusions of this article will be made available by the authors, without undue reservation.

## Footnotes

1 The original quotes are in the Greek language; therefore, there is a broad translation of participants’ words cited by their unique reference number (e.g., P14, P47).
